# The Impact of Face and Neck Burns on Respiratory Complications and Mortality

**DOI:** 10.3390/ebj6020027

**Published:** 2025-05-22

**Authors:** Rares-Adrian Giurgiu, Eliza-Maria Bordeanu-Diaconescu, Andreea Grosu-Bularda, Adrian Frunza, Sabina Grama, Raducu-Andrei Costache, Carina-Ioana Cristescu, Tiberiu-Paul Neagu, Ioan Lascar, Cristian-Sorin Hariga

**Affiliations:** 1 Department 11, Discipline Plastic and Reconstructive Surgery, University of Medicine and Pharmacy Carol Davila, 050474 Bucharest, Romania; rares.giurgiu@drd.umfcd.ro (R.-A.G.);; 2 Burn Centre, Emergency Clinical Hospital of Bucharest, 014461 Bucharest, Romania

**Keywords:** facial burns, inhalation injury, respiratory infections, burn infection

## Abstract

Face and neck burns present significant clinical challenges due to their proximity to the airway, predisposing patients to inhalation injuries and subsequent respiratory complications. In our cohort of 206 patients, facial and neck burns were associated with a markedly higher incidence of inhalation injury (34.8% vs. 2.8%), necessitating more frequent endotracheal intubation (51.9% vs. 14.1%). Furthermore, respiratory infections were significantly more common in patients with facial and neck burns (26.7% vs. 7%, *p* < 0.001), with respiratory secretion cultures revealing a predominance of Pseudomonas aeruginosa (39.58%), Acinetobacter baumanii (18.75%), and Klebsiella pneumoniae (6.25%). In contrast, patients without facial and neck burns primarily exhibited Pseudomonas aeruginosa (50%) in their cultures. These complications translated into a significantly increased mortality rate in patients with facial and neck burns (31.1% vs. 12.7%), with a reduced mean survival period (66.7 days vs. 84.3 days) and a 2.8-fold increase in the hazard of mortality. Additionally, older age emerged as a significant determinant for the development of respiratory infections. Multivariable model regression analysis revealed that only TBSA remained a consistent and independent predictor for adverse respiratory outcomes and increased mortality, while face and neck burns are more causally associated with TBSA.

## 1. Introduction

Burn injuries represent a significant global health burden, affecting millions of individuals annually, with varying degrees of morbidity and mortality. The severity of a burn injury is determined by multiple factors, including the depth (ranging from superficial to full-thickness burns), total body surface area (TBSA) affected, age of the patient, and presence of inhalation injury or associated trauma [1]. According to the World Health Organization (WHO), burns account for approximately 180,000 deaths annually, with the highest incidence occurring in low- and middle-income countries, where access to specialized burn care is limited [2]. Mortality rates are significantly influenced by the extent of burns, with TBSA involvement above 40% being associated with a high risk of death, especially in elderly patients and those with comorbidities [3]. Inhalation injury further increases mortality, often leading to respiratory complications and sepsis, which are the leading causes of death in burn patients [4].

Most facial burns are classed as partial-thickness and require hospital care due to challenges in wound management, including pain and infection prevention [5,6,7]. Full-thickness burns are less frequent, as the face′s rich vascular network facilitates rapid heat dissipation. However, when they occur, they tend to cause hypertrophic scarring and need skin grafting using allografts. While most facial burns result from flash injuries and are partial-thickness, full-thickness burns are more common in flame and contact burns [7,8,9].

Facial and neck burns are among the most challenging injuries due to their impact on essential functions such as breathing, vision, and oral competence, as well as their profound psychological effects. Burns in these areas frequently result from exposure to flame, scalds, or chemical and electrical injuries, with inhalation injury posing a significant risk due to airway edema and potential respiratory compromise [10] (pp. 550–570).

Most of the time, patients with face and neck burns (further referred to as FNBs) also present with inhalation injury, which is a predisposing factor to pneumonia, respiratory insufficiency, and death [11,12,13]. Inhalation injury occurs due to the inhalation of hot gases, smoke, and toxic combustion products, leading to thermal injury of the upper airway, chemical irritation, and systemic toxicity from substances such as carbon monoxide and cyanide [14]. Damage to the airway mucosa, through various mechanisms like direct cellular damage, the disruption of mucociliary clearance, airway obstruction, and proinflammatory cytokines, results in edema, airway obstruction, and impaired ciliary function, increasing the risk of respiratory distress, prolonged mechanical ventilation, and death [10] (pp. 580–600) and [15].

Patients with face and neck burns often need to be intubated for airway management in the prehospital setting [12,16,17], and this has proven to be an independent risk factor for developing pneumonia during hospitalization [11].

Among the patients who survive the initial 72 h after a burn injury, the most common cause of death is infection [18]. Pneumonia is the cause of death in almost half of the infection-related deaths in burnt patients [19]. Around half of the patients admitted to burn units present with face and neck burns [20], and the possible complications that result from this injury double their respiratory risk [21]; hence, the importance of this type of injury is very high.

Effective strategies for preventing infections in burn patients include early airway assessment, antiseptic wound care, early excision and grafting, infection control measures, timely weaning from mechanical ventilation, the avoidance of unnecessary intubation, and aggressive pulmonary hygiene [22,23].

This study aims to evaluate the impact of facial and neck burns on respiratory complications and mortality in burn patients. Specifically, we sought to compare clinical outcomes, including the incidence of inhalation injury, respiratory infections, and overall survival, between patients with and without facial and neck burns and to identify whether burn localization serves as an independent risk factor for adverse respiratory outcomes and increased mortality.

## 2. Materials and Methods

We retrospectively reviewed the hospital records of the burn patients admitted to the Burn Center of Bucharest Emergency Clinical Hospital over 2 years, between 1 January 2022 and 31 December 2023. Patients transferred to a different hospital in less than 48 h were excluded from the study. The following data were collected from the patients’ medical records: age, sex, % total body surface area (TBSA) burned, etiology and mechanism of the burn, burn depth, patients’ comorbidities, length of stay, survival, presence of face and neck burns, presence of inhalation injury, characteristics of airway management, results of bronchoscopy, the occurrence of pneumonia, and the results of microbiological cultures.

In order to assess the presence and the severity of inhalation injury, diagnostic tools like bronchoscopy, arterial blood gas analysis, and chest imaging were employed. All patients admitted to the burn unit underwent microbiological assessment at admission and periodically during hospitalization, depending on clinical suspicion and risk factors. The clinical signs, with X-ray imaging and the microbiological analysis from samples collected using swab cultures from the burn wounds, sputum cultures, endotracheal aspirate, and blood cultures, were correlated in order to diagnose and isolate the microbiological pathogen agent of pneumonia. Respiratory infection was defined by the presence of at least two of the following: a new and persistent infiltrate, consolidation, or cavitation on chest X-ray or CT scan; sepsis or a recent change in sputum character or purulence; and the identification of a pathogen, with a ≥10⁵ CFU/mL in tracheal aspirate or ≥10⁴ CFU/mL in bronchoalveolar lavage [5].

All the data from the study were analyzed using IBM SPSS Statistics 25 and illustrated using Microsoft Office Excel/Word 2024. The effects of each variable were calculated as hazard ratios with 95% confidence intervals along significance values. Models were tested for goodness-of-fit and significance values. The threshold considered for the significance level for all tests was α = 0.05.

This study received approval from the hospital′s ethics committee and was conducted following all the principles outlined in the Declaration of Helsinki.

## 3. Results

Our study included 206 patients, of whom 71 (34.5%) did not have face and neck burns (further referred to as nFNBs), while 135 (65.6%) presented with face and neck burns (FNBs). Most of the patients were men (140 patients—68%), without a significant differences between groups (*p* = 0.117); the mean age was 53.35 ± 18.08 years, with a median of 53 years (IQR = 39–65.25 years), with age being significantly lower in patients with face and neck burns (median = 49 years, IQR = 37–64 years) in comparison to patients without face and neck burns (median = 57 years, IQR = 45–71 years) (*p* = 0.013). The distribution of age categories between groups showed that younger patients (18–39 years) were significantly (*p* = 0.060) more likely to be associated with FNBs (31.1% vs. 18.3%) (Table 1).

The main comorbidities of the patients are presented in Table 2.

**Figure 1:**
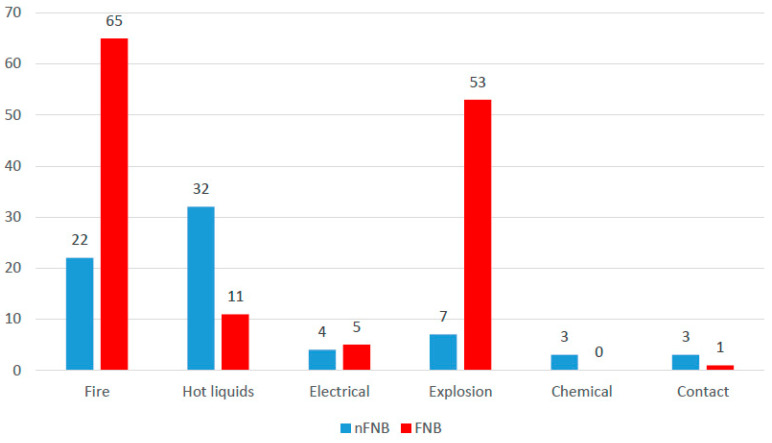
Distribution of patients according to burn etiology.

According to the burn etiology (Figure 1), most of the patients had burns because of fire (87 patients—42.2%), explosions (60 patients—29.1%), or hot liquids (43 patients—20.9%); the differences between groups were significant (Fisher’s exact test—*p* < 0.001), and Z-tests with Bonferroni correction showed that patients with fire burns (65 patients—48.1% vs. 22 patients—31%) or burns from explosions (53 patients—39.3% vs. 7 patients—9.9%) were significantly more associated with face and neck burns, while patients with burns from hot liquids (32 patients—45.1% vs. 11 patients—8.1%) or chemical burns (three patients—4.2% vs. zero patients—0%) were significantly less associated with face and neck burns.

**Figure 2:**
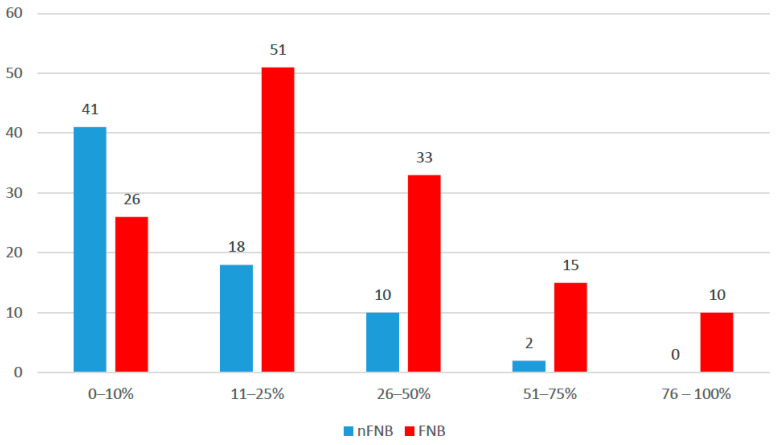
Distribution of patients according to TBSA.

The mean TBSA affected by burns was 25.36 ± 22.59%, with a median of 15% (IQR = 10–40%); patients with face and neck burns had a significantly higher TBSA burned (median = 20%, IQR = 12–45%) in comparison to patients without face and neck burns (median = 10%, IQR = 5–20%) (*p* < 0.001). According to burned TBSA categories (Figure 2), most of the patients had between 0 and 10% TBSA burned (67 patients—32.5%) or between 11 and 25% TBSA burned (69 patients—33.5%); the differences between groups were significant (Fisher’s exact test—*p* < 0.001), and Z-tests with Bonferroni correction showed that patients with burns affecting 0–10% of TBSA (41 patients—57.7% vs. 26 patients—19.3%) were significantly less associated with face and neck burns, while patients with burns affecting 51–75% of TBSA (15 patients—11.1% vs. 2 patients—2.8%) or 76–100% of TBSA (10 patients—7.4% vs. 0 patients—0%) were significantly more associated with the presence of face and neck burns (Figure 2).

The burn depth distribution of the groups is illustrated in Table 3, showing insignificant differences in burn degree categories between the groups (*p* = 0.239), with most of the patients having second-degree burns (47.9%—nFNB, 37.8%—FNB) or second- to third-degree burns (46.5%—nFNB, 52.6%—FNB).

Out of the 206 patients in the study group, 49 patients (23.8%) had inhalation injuries, almost all of whom were patients with face and neck burns (47 patients—34.8% with face and neck burns vs. 2 patients—2.8% without face and neck burns, Fisher’s exact test—*p* < 0.001). In our study group, 80 patients (38.83%) were intubated and mechanically ventilated.

The data indicate that patients with face and neck burns (FNBs) have a significantly higher risk of developing respiratory infections than those without such burns (nFNBs). In the group of patients without face and neck burns, 93% of patients did not develop a respiratory infection, while only 7% did. In contrast, in the group of patients with face and neck burns, 73.3% of patients did not develop a respiratory infection and 26.7% did, which constituted a statistically significant difference (*p* < 0.001). The rate of respiratory infections was not significantly associated with gender (*p* = 0.713); however, patients with respiratory infections were significantly older (median = 59, IQR = 48–74 vs. median = 52, IQR = 37–64.5, *p* = 0.010) than patients without respiratory infections, with patients aged between 18 and 39 years being significantly less associated with a respiratory infection (30.3% vs. 12.2%) and patients aged ≥65 years being significantly more associated with a respiratory infection (41.5% vs. 24.8%) (*p* = 0.022).

Patients with respiratory infections had a significantly higher TBSA (median = 45, IQR = 25–59 vs. median = 15, IQR = 8–25, *p* < 0.001) than patients without respiratory infections; patients with a lower TBSA, between 0 and 10%, were significantly less associated with respiratory infection (39.4% vs. 4.9%), while patients with a TBSA between 26 and 50% (34.1% vs. 12.7%) or between 51 and 75% (29.3% vs. 7.9%) were significantly more associated with respiratory infection (*p* < 0.001). Inhalation injury was also significantly more associated with respiratory infection (70.7% vs. 12.1%, *p* < 0.001) (Table 4).

The data presented in Table 5 show logistic binomial regression models that use TBSA and the presence of FNBs for the prediction of respiratory infections. Due to the linearity assumption invalidation using TBSA, a base-10 logarithm transformation was applied to TBSA, and the new variable was included in the models. According to the results, both TBSA and the presence of FNB are significant predictors of the existence of respiratory infections (*p* < 0.05) in the univariable models. In the multivariable model, the results show that the presence of face and neck burns is not a significant predictor (*p* = 0.152); only TBSA remains a significant predictor (*p* < 0.001); each 10-fold increase in the TBSA increases the odds of respiratory infections by 19.769 times (95% C.I.: 5.729–68.218).

The distribution of FNB groups according to the ETI scene shows significant differences (*p* < 0.001), and Z-tests with Bonferroni correction show that patients who were intubated at the scene of the burn injury were significantly more associated with FNBs (80% vs. 10%), while patients who were intubated in the emergency room (80% vs. 20%) or burn unit (10% vs. 0%) were significantly less associated with FNBs. Also, the mean ETI duration was 316.84 ± 401.7 h, with a median of 195 h (IQR = 100–392.5) and insignificant differences between FNB groups (*p* = 0.286) (Table 6).

Summary respiratory and blood isolates data are presented in Table 7. In nFNB isolates and respiratory secretions, only six cases of microorganisms were observed (one case of *Acinetobacter baumannii*, one case of *Corynebacterium striatum*, one case of *Klebsiella pneumoniae*, and three cases of *Pseudomonas aeruginosa*), while, in blood cultures, only six cases of microorganisms were observed (one case of *Escherichia coli*, one case of *Klebsiella oxytoca*, one case of *Klebsiella pneumoniae*, one case of *Providencia stuartii*, and two cases of *Pseudomonas aeruginosa*). In the case of FNB isolates and respiratory secretions, the most frequent microorganisms were *Pseudomonas aeruginosa* and *Acinetobacter baumannii*, while, in blood cultures, the most frequent microorganisms were *Pseudomonas aeruginosa*, *Acinetobacter baumannii*, and *Klebsiella pneumoniae*. Of the 41 patients diagnosed with bronchopneumonia, 27 were already intubated at the time of diagnosis; however, this finding did not reach statistical significance.

Mortality was significantly more associated with facial and neck burns (31.1% vs. 12.7%, *p* = 0.004), with the survival curve demonstrating a significantly lower overall survival period (mean = 66.781 days, 95% C.I.: 52.420–81.142) in comparison to patients without facial and neck burns (mean = 84.361 days, 95% C.I.: 68.404–100.317) (*p* = 0.003) (Table 8). An univariable Cox proportional-hazard regression model showed that the existence of facial and neck burns increased the hazard of mortality by 2.812 times (95% C.I.: 1.366–5.789) (*p* = 0.005). When adjusting for age and gender as potential mortality factors, the effect remained the same (HR = 3.602, 95% C.I.: 1.730–7.497, *p* = 0.001), with gender having an insignificant impact (HR = 1.050, 95% C.I.: 0.788–1.398, *p* = 0.741) and age having a significant impact on mortality; each increase of a year in the patients’ age increased their hazard of mortality by 1.032 times (95% C.I.: 1.014–1.049, *p* < 0.001).

Data from Table 9 show the Cox proportional-hazard regression models using TBSA, age, and the presence of FNBs. According to the results, TBSA, an age over or equal to 65 years, and the presence of FNBs are significant predictors of mortality (*p* < 0.05) in the univariable models. In the multivariable model, the results show that the presence of FNBs is not a significant predictor (*p* = 0.123); only TBSA (*p* < 0.001) and an age over or equal to 65 years (*p* < 0.001) remain significant predictors. Each increase in one unit of TBSA increases the risk of death by 1.052 times (95% C.I.: 1.039–1.066) and a patient’s age being equal or over 65 years increases the risk of death by four times (95% C.I.: 2.218–7.213).

## 4. Discussion

Facial burns are very common, accounting for 30–50% of minor to moderate burns and occurring in over half of severe burn cases [6]. Overall, the reported incidence rates vary widely in the literature, with the head and neck region being affected by burns in one-third to two-thirds of cases [20,24,25,26,27]. In our study, 65.6% of patients presented face and neck burns, a result similar to those found in a study by Tian et al. [27].

The majority of the patients with face and neck burns in our study were men, accounting for 140 individuals (68%). Although the proportion of male patients was higher among those with face and neck burns (97 patients, 71.9%) compared to those without face and neck burns (43 patients, 60.6%), this difference was not statistically significant, suggesting that gender was not a determining factor for the presence of face and neck burns in this study population. Patients with burns on the face and neck were notably younger, with a median age of 49 years (IQR = 37–64 years), compared to a median age of 57 years (IQR = 45–71 years) among those without face and neck burns (*p* = 0.013). This statistically significant difference suggests that younger individuals may be more susceptible to sustaining burns involving the face and neck, potentially due to increased exposure to risk-related activities, occupational hazards, or behavioral patterns common among younger demographics.

In our study, flame burns were more likely to present with face and neck involvement, with 65 patients (48.1%) in the group of patients with face and neck burns compared to 22 patients (31%) in the group of patients without face and neck burns. Similarly, flash burns were significantly associated with face and neck burns, affecting 53 patients (39.3%) compared to only 7 patients (9.9%) without face and neck burns. In contrast, scalds were significantly less associated with face and neck involvement, with only 11 patients (8.1%) in the face and neck group compared to 32 patients (45.1%) in the group of patients without face and neck burns. A similar trend was observed with chemical burns. These findings suggest that high-energy trauma mechanisms, such as fire and gas explosions, are more likely to result in face and neck burns, possibly due to the widespread nature of the thermal injury and the exposed position of these anatomical regions during such incidents. Conversely, scalds and chemical burns result in localized exposures that appear to spare the face and neck more frequently.

The mean total body surface area (TBSA) affected by burns across our study population was 25.36 ± 22.59%, with a median of 15% (IQR = 10–40%). However, patients with face and neck burns had significantly larger burn areas than those without face and neck burns. These findings suggest that the extent of burn injury is strongly correlated with the presence of face and neck involvement. A larger TBSA not only reflects more severe trauma but also likely increases the likelihood of facial exposure during injury events, such as house fires or LPG tank explosions.

The initial evaluation of burns in the face and neck focuses on assessing the airway and determining the burn depth. The primary concern in the initial management of facial and neck burns is airway stabilization due to the risk of upper airway edema and smoke inhalation injury. Assessing the need for intubation is mandatory both in the prehospital setting and upon arrival at the emergency department or burn center. Once the airway evaluation is complete, the initial treatment of facial and neck burns focuses on local wound care, including debridement and the application of topical antibiotics [28,29].

Inhalation injury is a significant contributor to morbidity and mortality in burn patients, often occurring alongside thermal injuries to the face and neck [30,31]. Unlike cutaneous burns, inhalation injuries affect the respiratory tract and systemic physiology through multiple mechanisms, including direct thermal damage, chemical irritation, and systemic toxicity from inhaled gases. These injuries can rapidly lead to respiratory distress, impaired oxygenation, and increased susceptibility to pulmonary complications such as pneumonia and acute respiratory distress syndrome (ARDS). The pathophysiology of inhalation injury involves three primary components. Supraglottic thermal injury occurs when hot gases cause burns to the upper airway, leading to mucosal edema and potential airway obstruction. In contrast, subglottic and alveolar injury results from the inhalation of toxic combustion byproducts, which can cause bronchospasm, inflammation, and surfactant dysfunction, impairing gas exchange. Additionally, systemic poisoning from absorbed small-molecule toxins—such as carbon monoxide and hydrogen cyanide—can lead to metabolic acidosis, tissue hypoxia, and multi-organ dysfunction [14,32,33,34,35]. Inhalation injuries are associated with cutaneous burns in up to 43% of cases, increasing mortality by up to 20% compared to patients presenting only with skin burns [36]. Inhalation injury predisposes patients to respiratory failure, acute respiratory distress syndrome, and pneumonia. In combination with inhalation injuries, pneumonia increases the mortality rate of these patients to 60% and is a risk factor for the development of acute respiratory distress syndrome [33]. Airway burns are associated with an increased risk of death, even in patients with a small burned body surface area [37].

In our study, inhalation injuries were observed in 49 patients (23.8%) across the study population, with a striking predominance among those with face and neck burns. Nearly all cases of inhalation injury occurred in the face and neck burns group, affecting 47 patients (34.8%) compared to only 2 patients (2.8%) in the group of patients without face and neck burns. This strong association highlights the vulnerability of the respiratory tract in patients with facial burns, likely due to direct exposure to heat, smoke, and toxic fumes during burn incidents. The presence of facial burns can therefore serve as a critical clinical indicator of the need for early airway assessment and potential intervention, including intubation and bronchoscopy, to prevent respiratory complications. Endotracheal intubation was required in 80 patients (38.8%) across the study cohort, with a significantly higher prevalence among those with face and neck burns. Specifically, 70 patients (51.9%) in the face and neck burns group underwent intubation, compared to only 10 patients (14.1%) in the group of patients without face and neck burns, a statistically significant difference. This finding highlights the critical impact of face burns on airway management, being often associated with upper airway edema, soft tissue inflammation, and potential inhalation injury, all of which can precipitate respiratory compromise.

Recent studies have questioned the predictive value of traditional intubation criteria for inhalation injury, such as facial burns, carbonaceous sputum, and singed nasal hair, due to their poor correlation with airway edema. A retrospective review of 47 patients undergoing bronchoscopy found these findings unreliable as absolute indicators for intubation [38]. Another study using flexible fiberoptic laryngoscopy in burn patients suggested that direct airway examination could help avoid unnecessary intubation [39]. As a result of these findings, the researchers proposed what became known as the Denver Criteria, a revised approach for assessing the need for intubation in burn patients. These criteria emphasized clinical signs such as respiratory distress, airway swelling seen on laryngoscopy, upper airway trauma, and hemodynamic instability. The study concluded that patients who met these criteria should be strongly considered for intubation, while those who did not could potentially be managed through careful observation rather than immediate airway intervention [40]. In our study, among the patients with face and neck burns, 70 patients were intubated and mechanically ventilated, although bronchoscopy confirmed an inhalation injury in only 29 cases. This discrepancy suggests that clinical indicators alone may have prompted early airway management, potentially leading to an overestimation of inhalation injury in the acute setting.

In our study, in the univariate analysis, respiratory infection was significantly more common in patients with FNBs (26.7% vs. 7%, *p* < 0.001), likely due to a combination of direct airway injury, impaired mucociliary clearance, increased secretions, and prolonged ventilatory support. In patients with face and neck burns, respiratory secretion cultures most frequently isolated Pseudomonas aeruginosa (39.58%), Acinetobacter baumanii (18.75%), and Klebsiella pneumoniae (6.25%), while, in patients without face and neck burns, Pseudomonas aeruginosa was the predominant pathogen (50% of cases). In a similar study conducted by Costa Santos et al., the predominant respiratory pathogens among patients with facial and neck burns were Staphylococcus aureus (20%), followed by Pseudomonas aeruginosa (15%) and Streptococcus pneumoniae (10%), whereas, in patients without such burns, Klebsiella pneumoniae was the most frequently isolated strain (33.3%) [12].

Patients with face and neck burns are at high risk for infections, particularly early-onset pneumonia (EOP), due to airway injury, mechanical ventilation, and immune suppression. A study by Cotte et al. [41] found that 38.2% of patients with severe facial burns developed early-onset pneumonia, with prehospital intubation being the strongest risk factor. Other risk factors include inhalation injury, a large burn surface area, and impaired respiratory clearance. For EOP, the predominant organisms include *Staphylococcus aureus* (56.9%), *Streptococcus pneumoniae* (19%), and *Haemophilus influenzae* (19%) [41]. For ventilator-associated pneumonia (VAP) or late-onset pneumonia, nosocomial pathogens such as *Pseudomonas aeruginosa*, *Klebsiella pneumoniae*, and multidrug-resistant (MDR) *Enterobacteriaceae* become more prevalent [42,43].

Burn-related pneumonia significantly prolongs hospital stays, increases ventilator dependency, and raises the risk of sepsis and organ failure. Studies indicate that burn patients with pneumonia have a two- to three-fold higher mortality rate than those without [44,45]. Inhalation injury further worsens pulmonary function, leading to respiratory distress syndrome and systemic inflammation, which contribute to multiorgan dysfunction syndrome (MODS) [10].

Our study showed a significant association in the univariate analysis between the burn surface area and the occurrence of respiratory infections. Patients who developed respiratory infections had a significantly higher median TBSA (45%, IQR: 25–59) than those without respiratory infections (median: 15%, IQR: 8–25, *p* < 0.001). These findings suggest that as burn severity increases, the likelihood of respiratory infections also rises, likely due to the increased inflammatory response, systemic immunosuppression, and prolonged need for mechanical ventilation and intensive care in more severely burned patients.

In the univariable analysis, both TBSA and FNBs were significantly associated with respiratory infections, suggesting that a larger burn extent and specific burn location may play a role in the development of pulmonary complications. However, in the multivariable model, only TBSA remained a significant predictor, whereas the presence of FNBs lost statistical significance. Notably, a 10-fold increase in TBSA was associated with nearly a 20-fold increase in the odds of respiratory infection. The lack of an independent association between FNBs and respiratory infections in the adjusted model may reflect confounding by burn severity or the potential for airway involvement to be better captured by direct inhalation injury assessment rather than anatomical burn location alone.

In our study, age was another significant determinant for respiratory infections, with older patients being at greater risk for respiratory infections. Patients with infections had a higher median age (59 years, IQR = 48–74) than those without infections (52 years, IQR = 37–64.5; *p* = 0.010). Age-group analysis further confirmed that young adults (18–39 years) were significantly less likely to develop respiratory infections (30.3% vs. 12.2%), whereas elderly patients (≥65 years) were significantly more affected (41.5% vs. 24.8%; *p* = 0.022). This association can be attributed to age-related immune decline, a reduced pulmonary reserve, and the presence of chronic conditions that may impair recovery from pulmonary insults.

The mortality in our cohort in the univariate analysis was significantly higher in patients with facial and neck burns than in those without these injuries, with rates of 31.1% versus 12.7%. The survival analysis reinforces this observation, showing that patients with facial and neck burns had a notably shorter overall survival period, with a mean survival of 66.7 days compared to 84.3 days in patients without these burns. The presence of facial and neck burns increased the hazard of mortality by 2.8 times in our study group. The Cox proportional-hazard regression analysis provided important insights into the factors influencing mortality in burn patients. Univariable models identified TBSA, age ≥ 65 years, and the presence of face and neck burns as significant predictors of mortality. However, in the multivariable model, only TBSA and an age ≥65 years remained statistically significant, while FNBs were no longer an independent predictor of mortality, aligning with established evidence that the extent of the burn and an advanced age may adversely affect survival.

## 5. Conclusions

Our study highlights that facial and neck burns represent a particularly severe subset of injuries and are associated with larger burn areas, a higher incidence of inhalation injury, and increased rates of respiratory infections. Patients with face and neck burns have distinct microbiological profiles in respiratory secretion cultures and face significantly higher mortality rates than those without this type of burn. While both TBSA and the presence of face and neck burns were initially associated with respiratory infections and mortality in univariable analyses, multivariable models revealed that only TBSA remained a consistent and independent predictor in both outcomes. Additionally, being aged ≥ 65 years emerged as a strong predictor of mortality, further emphasizing the vulnerability of elderly patients.

## Figures and Tables

**Table 1:** Demographic characteristics of the analyzed patients.

Parameter	nFNB (n = 71)	FNB (n = 135)	*p*
Gender (male) (Nr., %)	43 (60.6%)	97 (71.9%)	0.117
Age (median (IQR))	57 (45–71)	49 (37–64)	0.013
Age category (Nr., %)			
18–39 years	13 (18.3%)	42 (31.1%)	0.060
40–64 years	32 (45.1%)	61 (45.2%)	
≥65 years	26 (36.6%)	32 (23.7%)	

**Table 2:** Distribution of FNB groups according to medical history.

Medical Condition (Nr., %)	nFNB (n = 71)	FNB (n = 135)
Hypertension	32 (45.1%)	30 (22.2%)
Other cardiovascular diseases	18 (25.4%)	22 (16.3%)
Diabetes mellitus	16 (22.5%)	11 (8.1%)
Neurologic disease	8 (11.3%)	15 (11.1%)
Asthma	2 (2.8%)	0 (0%)
COPD	2 (2.8%)	3 (2.2%)
Drug abuse	1 (1.4%)	1 (0.7%)
Psychiatric illness	4 (5.6%)	16 (11.8%)
Neurologic disease	8 (11.3%)	15 (11.1%)
Asthma	2 (2.8%)	0 (0%)
COPD	2 (2.8%)	3 (2.2%)

**Table 3:** Distribution of FNB groups according to burn depth.

Burn Degree (Nr., %)	nFNB (n = 71)	FNB (n = 135)	*p*
1st + 2nd	2 (2.8%)	11 (8.1%)	0.239
2nd	34 (47.9%)	51 (37.8%)	
2nd + 3rd	33 (46.5%)	71 (52.6%)	
3rd	2 (2.8%)	2 (1.5%)	

**Table 4:** Distribution of groups of patients according to respiratory infection and gender, age, and TBSA.

Parameter (Nr., %)	Without Infection	With Infection	*p*
Gender			
- Female	54 (32.7%)	12 (29.3%)	0.713
- Male	111 (67.3%)	29 (70.7%)	
Age (median (IQR))	52 (37–64.5)	59 (48–74)	0.010
Age			
18–39 years	50 (30.3%)	5 (12.2%)	0.022
40–64 years	74 (44.8%)	19 (46.3)%	
≥65 years	41 (24.8%)	17 (41.5%)	
TBSA (median (IQR))	15 (8–25)	45 (25–59)	<0.001
TBSA			
0–10%	65 (39.4%)	2 (4.9%)	<0.001
11–25%	60 (36.4%)	9 (22%)	
26–50%	21 (12.7%)	14 (34.1%)	
51–75%	13 (7.9%)	12 (29.3%)	
76–100%	6 (3.6%)	4 (9.8%)	

**Table 5:** Logistic binomial regression models using TBSA and presence of FNBs for the prediction of respiratory infections.

Dependent Variable = Respiratory Infections				
Parameter	Univariable		Multivariable	
Log10 (TBSA)	25.105 (7.574–83.215)	<0.001	19.769 (5.729–68.218)	<0.001
FNB	4.800 (1.791–12.865)	0.002	2.172 (0.751–6.281)	0.152

**Table 6:** Distribution of groups of patients according to endotracheal intubation.

Parameter (Nr., %)	nFNB (n = 71)	FNB (n = 135)	*p*
Inhalation injury	20 (12.1%)	29 (70.7%)	<0.001
Endotracheal intubation (ETI)	10 (14.1%)	70 (51.9%)	<0.001
ETI scene			
- Burn injury scene	1 (10%)	56 (80%)	
- Emergency room	8 (80%)	14 (20%)	<0.001
- Burn unit	1 (10%)	0 (0%)	

**Table 7:** Description of microbiological isolates in FNB groups.

*Species* (Nr., %)	nFNB Respiratory	FNB Respiratory	nFNB Blood	FNB Blood
*Acinetobacter baumannii*	1 (16.7%)	9 (18.75%)	-	4 (19.05%)
*Aspergillus* spp.	-	1 (2.08%)	-	-
*Burkholderia cepacia*		1 (2.08%)	-	-
*Candida albicans*	-	-	-	1 (4.76%)
*Candida parapsilosis*	-	-	-	2 (9.52%)
*Citrobacter* spp.		1 (2.08%)	-	-
*Corynebacterium striatum*	1 (16.7%)	2 (4.17%)	-	-
*Escherichia coli*	-	-	1 (16.7%)	1 (4.76%)
*Haemophylus influenzae*	-	1 (2.08%)	-	-
*Klebsiella aerogenes*	-	1 (2.08%)	-	-
*Klebsiella oxytoca*	-	-	1 (16.7%)	-
*Klebsiella pneumoniae*	1 (16.7%)	3 (6.25%)	1 (16.7%)	3 (14.29%)
*Proteus vulgaris*	-	2 (4.17%)	-	
*Providencia stuartii*	-	1 (2.08%)	1 (16.7%)	-
*Serratia marcescens*	-	-	-	1 (4.76%)
*Pseudomonas aeruginosa*	3 (50%)	19 (39.58%)	2 (33.3%)	5 (23.81%)
*Pseudomonas putida*	-	2 (4.17%)	-	-
*Serratia marcescens*	-	2 (4.17%)	-	-
*Staphylococcus aureus*	-	-	-	1 (4.76%)
*Staphylococcus haemolyticus*	-	-	-	2 (9.52%)
*Staphylococcus hominis*	-	-	-	1 (4.76%)
*Stenotrophomonas maltophilia*	-	3 (6.25%)	-	-
Total	6	48	6	21

**Table 8:** Distribution of FNB groups according to survival.

Mortality (Nr., %)	nFNB (n = 71)	FNB (n = 135)	*p*
Survivors	62 (87.3%)	93 (68.9%)	0.004 *
Deceased	9 (12.7%)	42 (31.1%)	
Overall survival (days)	84.361(68.404–100.317)	66.781 (52.420–81.142)	0.003 **
Mean (95% C.I.)			

* Fisher’s exact test; ** log-rank test.

**Table 9:** Cox proportional-hazard regression models using TBSA, age, presence of face and neck burns, and comorbidities for the prediction of mortality.

Dependent Variable = Respiratory Infections				
Parameter	Univariable		Multivariable	
TBSA	1.050 (1.037–1.062)	<0.001	1.052 (1.039–1.066)	<0.001
FNB	2.812 (1.366–5.789)	0.005	1.842 (0.848–4.002)	0.123
Age ≥ 65 years	2.294 (1.312–4.009)	0.004	4.000 (2.218–7.213)	<0.001

## Data Availability

Dataset available on request from the authors.
